# Identification of PDLIM1 as a glioblastoma stem cell marker driving tumorigenesis and chemoresistance

**DOI:** 10.1038/s41420-024-02241-7

**Published:** 2024-11-15

**Authors:** Xiaopeng Shen, Yun Zhao, Yang Cao, Yunfeng Liu, Jian Ruan, Chunguang Wang, Meng Li, Huaizhang Jin, Shan Lu, Guoping Zhu

**Affiliations:** 1 College of Life Sciences, Anhui Normal University, Wuhu, Anhui China; 2 Center of Reproductive Medicine, Yijishan Hospital of Wannan Medical College, Wuhu, Anhui China

**Keywords:** Prognostic markers, Tumour biomarkers

## Abstract

Glioblastoma (GBM) is an aggressive brain tumor with a poor prognosis, largely due to the presence of glioblastoma stem cells (GSCs). These cells drive tumor progression, recurrence, and chemoresistance, making them critical targets for therapy. This study aims to identify novel GSC markers for improved diagnosis and targeted treatment. We utilized single-cell RNA sequencing (scRNA-seq) and bulk RNA-seq data to identify PDLIM1 as a novel GSC marker. PDLIM1 was specifically expressed in GSCs and was associated with poor prognosis and advanced tumor stages. Functional assays demonstrated that PDLIM1 overexpression enhanced GBM cell proliferation, reduced apoptosis, increased GSC proportions, and promoted chemoresistance and tumorigenesis. Conversely, PDLIM1 knockdown inhibited these processes. Mechanistically, PDLIM1 was found to exert its effects likely by promoting the PI3K-AKT pathway. In conclusion, PDLIM1 may serve as a potential marker of GSCs associated with poor prognosis, tumorigenesis, and chemoresistance in GBM, representing a potential therapeutic target for improving GBM patient outcomes.

## Introduction

Glioblastoma (GBM) is a highly aggressive brain tumor that remains resistant to current therapeutic strategies. Despite advances in treatment, the median overall survival (OS) for GBM patients is still less than 15 months [1]. Standard treatment involves surgical resection followed by chemotherapy and radiotherapy, with temozolomide (TMZ) being the most commonly used chemotherapeutic agent [1]. Unfortunately, the prognosis for GBM remains poor due to several factors, including the presence of glioblastoma stem cells (GSCs) and the challenge of the blood-brain barrier. Emerging research indicates that GSCs play a crucial role in GBM progression and resistance to both chemotherapy and radiotherapy. Several pathways, such as PI3K/AKT [2], HIF-1α [3], and Wnt [4], are vital for GSC differentiation and maintenance. However, the precise regulatory mechanisms of these pathways in GSCs are not fully understood. Given the critical role of GSCs in GBM, significant efforts have been made to identify GSC-specific biomarkers, biological processes, and signaling pathways, and to develop corresponding intervention strategies. Currently identified GSC markers include PROM1, CD44, and SOX2 [5]. Nonetheless, further investigation is needed to discover additional markers that are specifically expressed in GSCs and that govern their stemness and chemoresistance.

PDLIM1 (also known as CLP36) is recognized as a key component in cytoskeleton organization and development. Comprising 329 amino acids, PDLIM1 contains a PDZ domain and a LIM domain, enabling it to serve as a scaffold for the formation of protein complexes and the regulation of cellular processes and signaling transductions [6]. PDLIM1 interacts with various α-actinin isoforms in different tissues or cells: it primarily interacts with α-actinin-1/4 in colonic epithelial cells [7, 8] and with α-actinin-2 in the myocardium [9]. Additionally, PDLIM1 interacts with palladin, thereby regulating nerve regeneration [10]. Dysregulation of PDLIM1 has been observed in multiple cancers, including colorectal cancer [10], hepatocellular carcinoma [11], breast cancer [12], and glioma [13]. In glioma, PDLIM1 has been identified as an adapter to the neurotrophin receptor p75NTR, mediating glioma invasion [13]. However, its roles in other aspects of tumorigenesis, prognosis, and GSCs in glioma and GBM remain unknown.

In this study, we analyzed single-cell RNA sequencing (scRNA-seq) data of GBM to identify novel markers for GSCs. By integrating expression patterns and prognostic impacts, we selected PDLIM1 as a potential GSC marker for further investigation. Through in vitro and in vivo experiments, we discovered that PDLIM1 significantly promoted GBM growth, suppressed apoptosis, and increased the proportion of GSCs among GBM cells, thereby enhancing GBM tumorigenesis and chemoresistance. Finally, we explored the effects of PDLIM1 on the PI3K/AKT pathway and found that PDLIM1 significantly activated this pathway, which might mediate its impact on GBM tumorigenesis and GSC activities.

## Results

### PDLIM1 was a novel glioblastoma stem cell marker associated with poor prognosis

Glioblastoma (GBM) is a highly malignant and recurrent tumor, partly due to the presence of glioblastoma stem cells (GSCs). GSCs are known to promote metastasis, recurrence, and chemoresistance in GBM [14–16]. Several GSC-related markers have been proposed, such as PROM1, CD44, and SOX2 [5]. However, identifying additional GSC markers is crucial for developing targeted therapeutic drugs. The advent of single-cell RNA sequencing (scRNA-seq) provides a powerful tool for identifying cell markers.

**Fig. 1:**
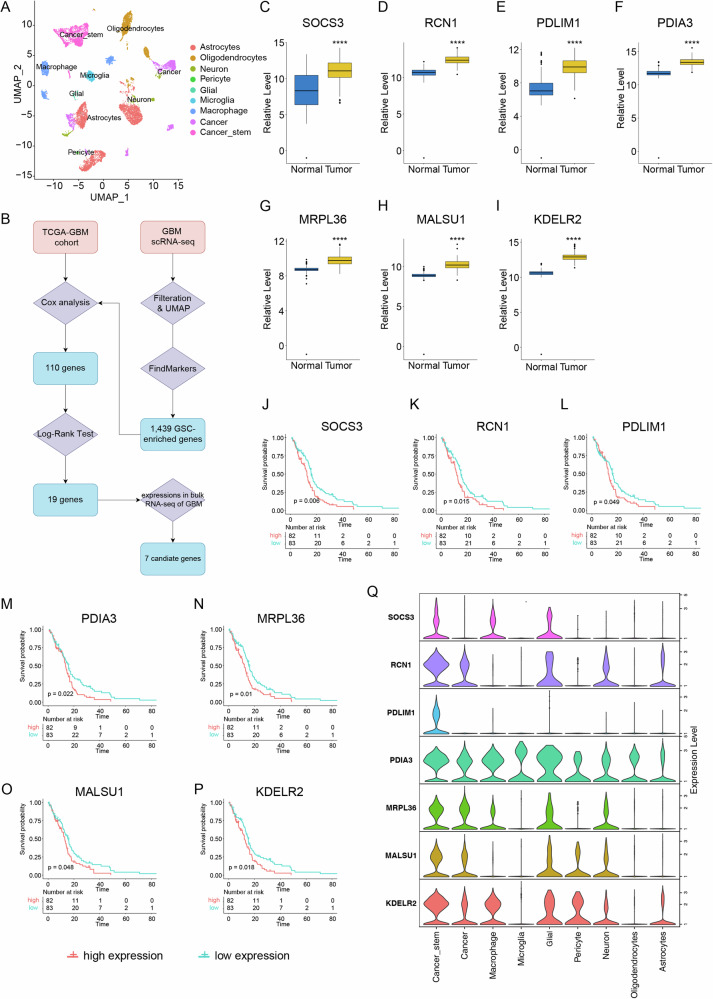
PDLIM1 was a novel glioblastoma stem cell marker associated with poor prognosis. **A** The UMAP plot showing the cell subgroups in the GBM scRNA-seq data. **B** The flowchart showing the process of identifying seven candidate genes. The relative levels of **C** SOCS3, **D** RCN1, **E** PDLIM1, **F** PDIA3, **G** MRPL36, **H** MALSU1, and **I** KDELR2 in GBM compared to normal brain tissues. The Kaplan-Meier overall survival (OS) curves between the patients of the TCGA-GBM cohort with high- and low-expressions of **J** SOCS3, **K** RCN1, **L** PDLIM1, **M** PDIA3, **N** MRPL36, **O** MALSU1, and **P** KDELR2, respectively. **Q** The relative levels of SOCS3, RCN1, PDLIM1, PDIA3, MRPL36, MALSU1, and KDELR2 in different cell subgroups in GBM scRNA-seq data. ****P < 0.0001.

In this study, we analyzed a scRNA-seq dataset of GBM, consisting of 12,259 cells from the tumor samples of nine patients post data filtration. Using previously reported markers, we annotated nine cell subgroups: astrocytes, oligodendrocytes, neurons, pericytes, glial cells, microglia, macrophages, cancer cells, and cancer stem cells (Fig. 1A). We identified potential novel marker genes of GBM cancer stem cells using the “FindMarkers” function in the “Seurat” R package, setting a significance threshold of |log2(FoldChange)| > 0.25 and *P* < 0.05. This analysis yielded 1439 marker genes for cancer stem cells (Fig. 1B).

Next, we conducted univariate Cox regression analyses on these genes in the TCGA-GBM cohort, identifying 110 genes significantly affecting GBM prognosis. Log-Rank survival analyses on these genes further revealed 19 significantly associated with overall survival (OS) in GBM (Fig. 1B). Among these, seven genes, namely SOCS3, RCN1, PDLIM1, PDIA3, MRPL36, MALSU1, and KDELR2, were significantly upregulated in GBM compared to normal brain tissues (Fig. 1C–I) and correlated with shortened OS (Fig. 1J–P). By re-analyzing scRNA-seq data, we found that among these genes, only PDLIM1 was specifically expressed in the cancer stem cells, although the other six genes showed some enrichment in cancer stem cells (Fig. 1Q). Thus, PDLIM1 might be a novel GSC marker significantly upregulated in GBM and associated with poor prognosis.

We further explored the interactions of PDLIM1 with all cell subgroups and their marker genes in GBM scRNA-seq data. Cancer stem cells were the only subgroup significantly correlated with PDLIM1 expression (Fig. S1A). Among the specifically expressed genes of each subgroup, PDLIM1 correlated only with three other cancer stem cell markers, namely PDGFRA, RBP1, and ITM2A (Fig. S1B). We examined the impacts of PDLIM1 on the expression of GSC-related genes in three independent GBM cohorts (TCGA-GBM, CGGA-693, and CGGA-325). The patients in each cohort were equally divided into the low- and high-PDLIM1 groups according to their corresponding median PDLIM1 expressions. In all three cohorts, GSC-related genes were upregulated in the high-PDLIM1 group compared to the low-PDLIM1 group, indicating that PDLIM1 was a specific GSC marker influencing the expressions of other GSC-related genes (Fig. S1C–E).

### PDLIM1 was associated with GBM progression and prognosis

**Fig. 2:**
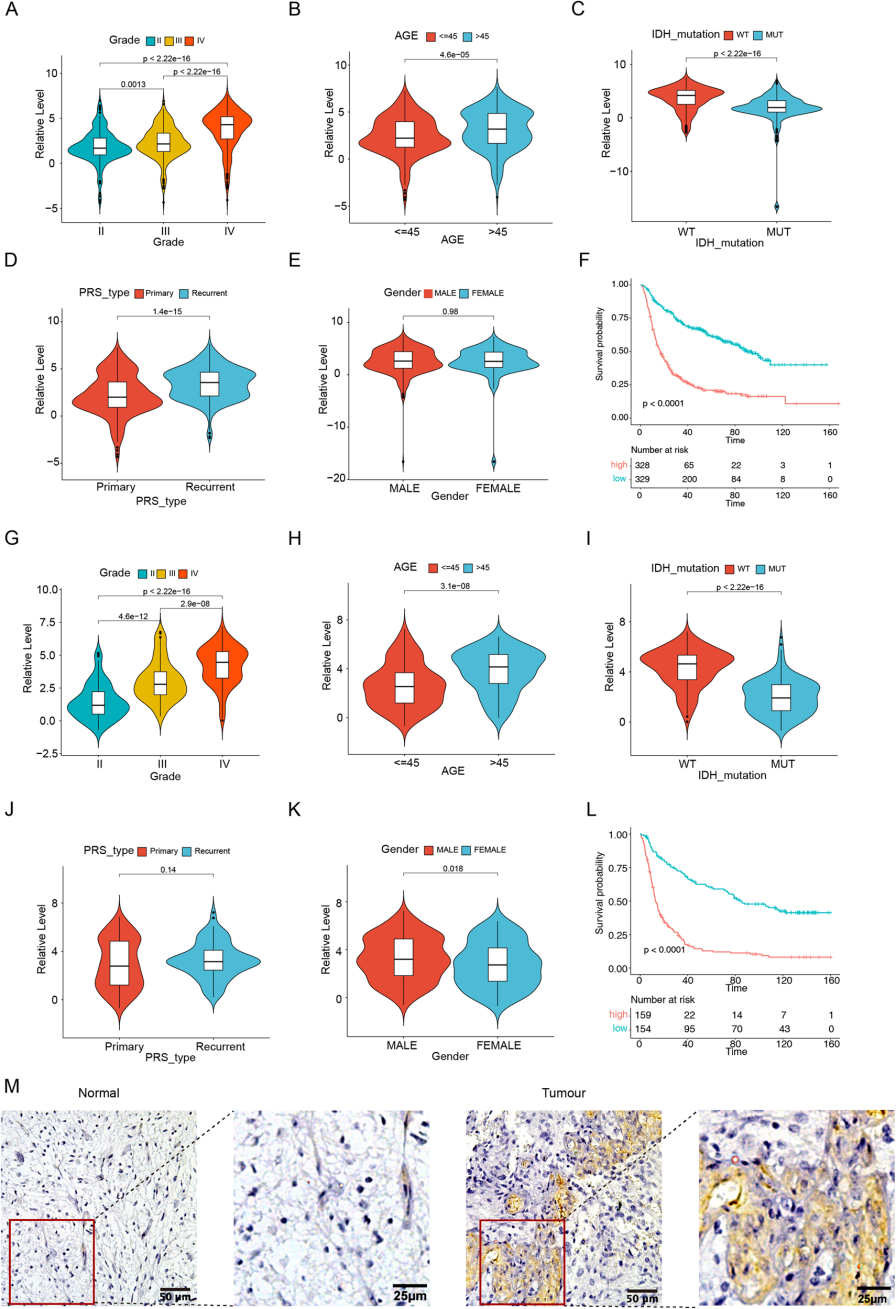
The expressions of PDLIM1 regarding different clinical parameters and the involvement of PDLIM1 in GBM OS. The relative levels of PDLIM1 regarding different **A** histological stages, **B** age, **C** IDH1 mutation status, **D** recurrence status, and **E** gender in the CGGA-693 cohort. **F** The Kaplan–Meier OS curve between the patients of the CGGA-693 cohort with high- and low-expressions of PDLIM1. The relative levels of PDLIM1 regarding different **G** histological stages, **H** age, **I** IDH1 mutation status, **J** recurrence status, and **K** gender in the CGGA-325 cohort. **L** The Kaplan–Meier OS curve between the patients of the CGGA-325 cohort with high- and low-expressions of PDLIM1. **M** The immunohistochemistry staining against PDLIM1 on the GBM and para-tumor normal brain tissue section slides.

We investigated the correlations of PDLIM1 expressions with other clinical parameters in GBM. In both the CGGA-693 and CGGA-325 cohorts, PDLIM1 expressions were significantly enhanced in the patients with advancing histological stages, older ages, and wild-type IDH1 (Fig. 2A–C, G–I). The expression changes regarding recurrence status and gender were inconsistent across the cohorts (Fig. 2D, E, J, K). In line with the TCGA-GBM cohort (Fig. 1L), both the CGGA-693 and CGGA-325 cohorts displayed significantly shortened OS with high-PDLIM1 expressions (Fig. 2F, L). Moreover, we performed immunohistochemistry staining against PDLIM1 on the GBM section slides along with the para-tumor normal brain tissue slides. As a result, we noticed evident expressions of PDLIM1 in GBM but no visible PDLIM1 staining in the normal group (Fig. 2M).

Next, we performed univariate and multivariate Cox regression analyses on PDLIM1 expression and other clinical parameters to assess the prognostic value of PDLIM1. We included recurrent status, gender, age, IDH1 mutation, histological stage, and PDLIM1 expression for the Cox regressions on the CGGA-693 and CGGA-325 cohorts, and gender, race, age, and PDLIM1 expression for the Cox regressions on the TCGA-GBM cohort. This bias was caused by the differences in the documented clinical information of these cohorts. Consequently, PDLIM1 expression was identified as a significant hazard factor in both univariate and multivariate Cox regressions across all three cohorts, indicating that PDLIM1 is an independent prognostic factor for GBM (Fig. S2A, B, D, E, G, H). We then divided each cohort into low- and high-risk groups based on their risk scores. The high-risk groups had a higher number of deaths and shorter overall survival (OS). PDLIM1 expression was notably upregulated in the high-risk groups, further confirming its critical prognostic value (Fig. S2C, F, I).

### PDLIM1 overexpression promoted GBM progression and GSC activities

**Fig. 3:**
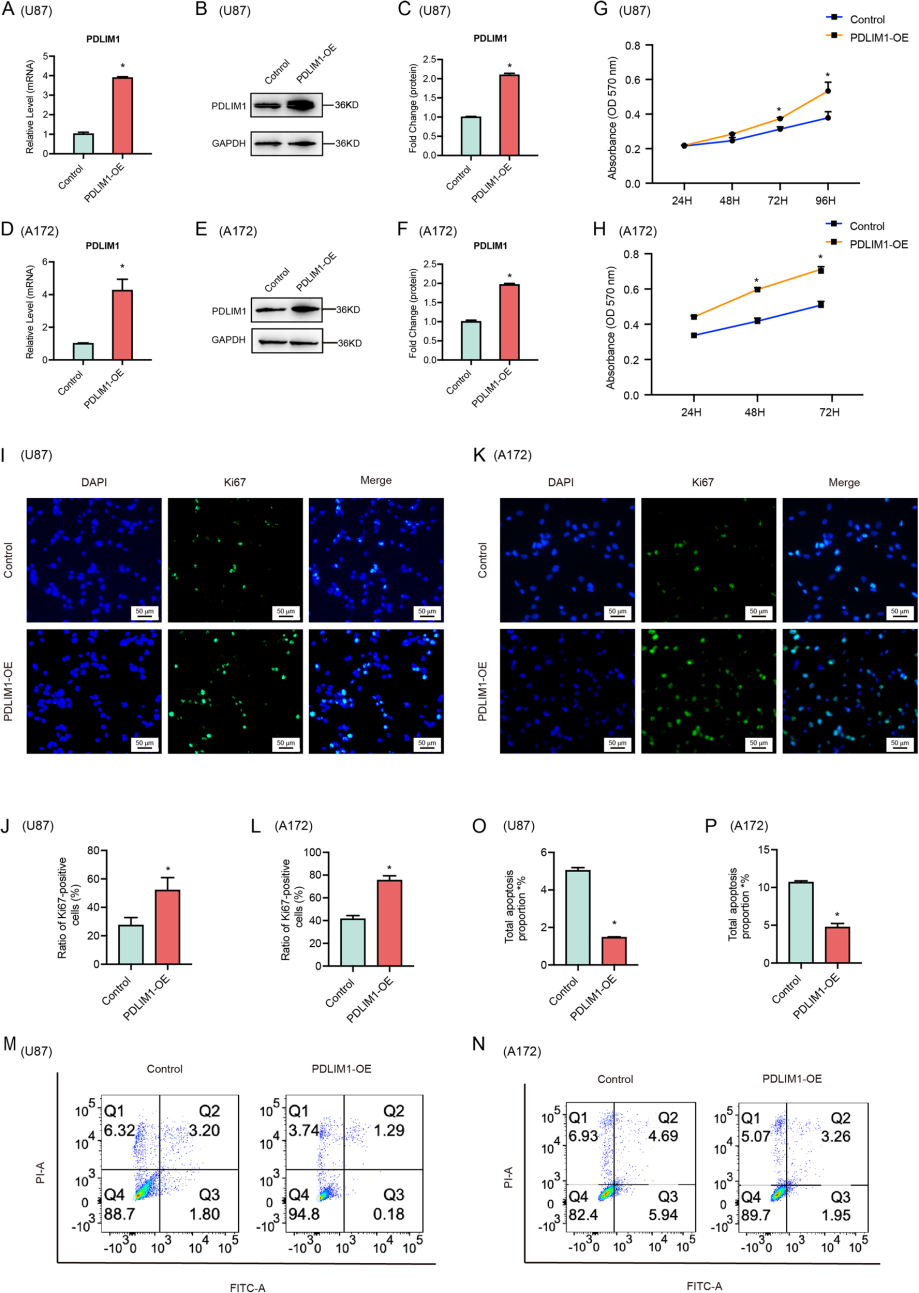
PDLIM1 overexpression promoted GBM progression. The overexpression of PDLIM1 in U87 cells was validated by **A** RT-qPCR and **B**, **C** western blots. The overexpression of PDLIM1 in A172 cells was validated by **D** RT-qPCR and **E, F** western blots. MTT assays showing that PDLIM1 overexpression promoted cell growth in both **G** U87 and **H** A172 cells, respectively. Immunostaining against Ki67 was performed on control and PDLIM1-overexpressing **I**, **J** U87 and **K, L** A172 cells, respectively. Annexin V/PI apoptosis assays were performed on control and PDLIM1-overexpressing **M**, **O** U87 and **N**, **P** A172 cells, respectively. Control, empty vector control; PDLIM1-OE, PDLIM1 overexpression; *P < 0.05.

To systematically study PDLIM1’s function in GBM, we constructed stable PDLIM1 overexpression (PDLIM1-OE) in U87 and A172 cells, respectively, using empty vectors as controls. Overexpression was validated by RT-qPCR and western blots (Fig. 3A–F). MTT assays showed a significant increase in cell growth with PDLIM1-OE in both cell lines (Fig. 3G, H). Immunostaining against Ki67 revealed a significant increase in Ki67+ cell proportion with PDLIM1-OE, indicating that PDLIM1-OE enhanced cell cycle activity (Fig. 3I–L). Annexin V/PI apoptosis assays demonstrated that PDLIM1-OE significantly reduced apoptosis in GBM cells (Fig. 3M–P).

**Fig. 4:**
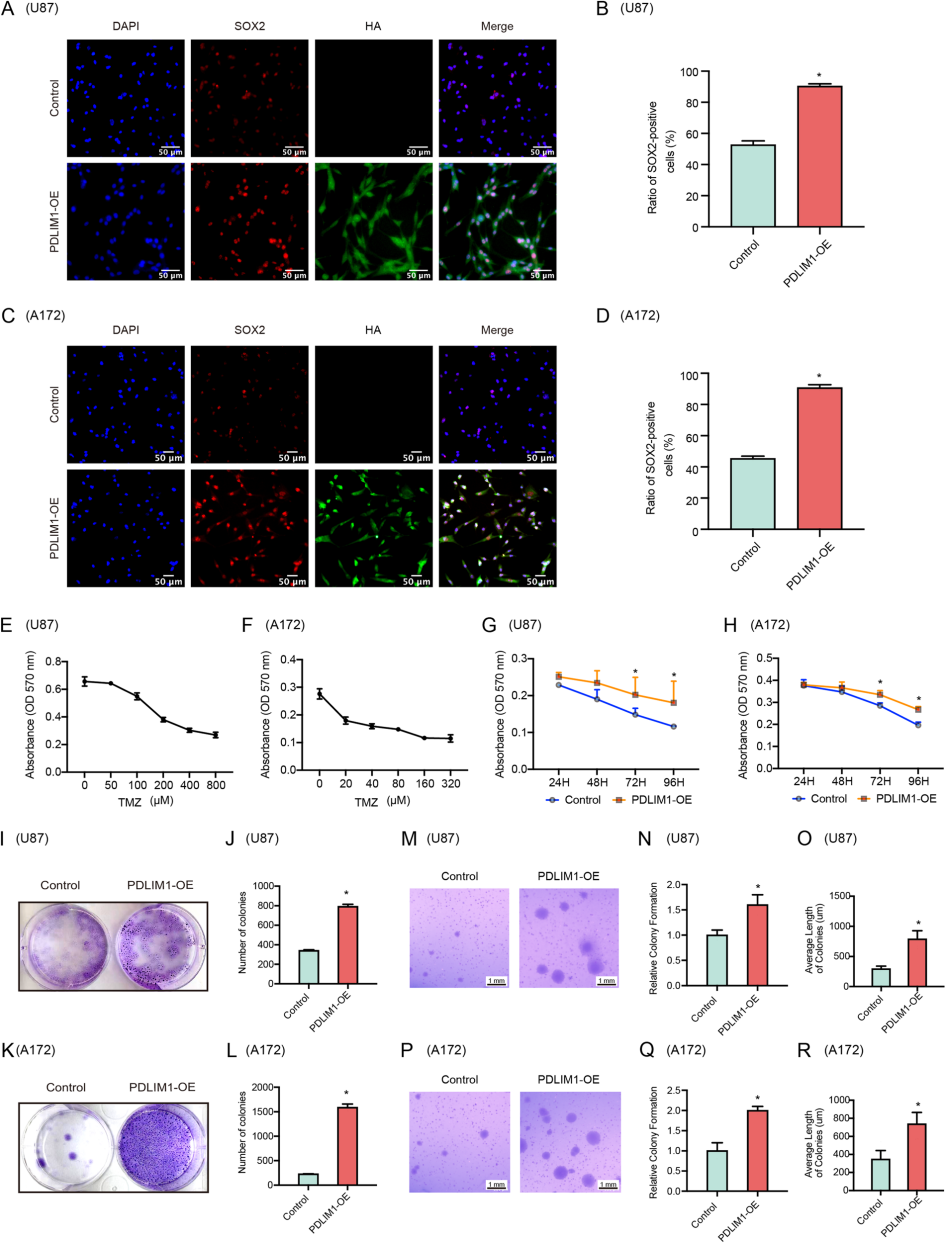
PDLIM1 overexpression promoted GSC-mediated processes. Co-immunostaining against HA-tagged PDLIM1 and SOX2 was performed on control and PDLIM1-overexpressing **A**, **B** U87 and **C**, **D** A172 cells, respectively. The IC50 of temozolomide (TMZ) was determined on **E** U87 and **F** A172 cells, respectively. The overexpression of PDLIM1 significantly improved the chemoresistance of **G** U87 and **H** A172 cells to TMZ. Colony formation assays were performed on control and PDLIM1-overexpressing **I**, **J** U87 and **K**, **L** A172 cells, respectively. Soft agar assays were performed on control and PDLIM1-overexpressing **M**–**O** U87 and **P**–**R** A172 cells, respectively. Control, empty vector control; PDLIM1-OE, PDLIM1 overexpression; *P < 0.05.

As PDLIM1 was proposed to be a potential GSC marker (Fig. 1Q), we wondered if PDLIM1-OE was able to improve the ratio of GSC in the GBM cell population. As the overexpressed PDLIM1 had a HA tag, we performed co-immunostaining against HA and a canonical GSC marker, SOX2, on PDLIM1-OE and control GBM cells. In control U87 and A172 cells, the SOX2 + GSC ratios were 52.67 ± 1.45% and 45.34 ± 0.88%, respectively. However, these ratios were boosted to 90.33 ± 0.88% and 90.66 ± 1.20% with PDLIM1-OE (Fig. 4A–D). These results suggested that PDLIM1 expressions tightly controlled the GSC ratio within GBM cells. As known to all, GSC was closely associated with the chemoresistance, tumorigenesis, and metastasis of GBM. We first evaluated the chemoresistance changes of GBM regarding PDLIM1-OE. Initially, we determined the IC50 of temozolomide (TMZ) on U87 and A172 cells, respectively. By MTT assays, we determined that the IC50 for U87 and A172 cells were approximal 400 And 80 μM, respectively (Fig. 4E, F). Subsequently, we treated U87 and A172 cells with TMZ at their corresponding IC50 concentrations and measured cell activities at different time points, respectively. We found that PDLIM1-OE eventually caused a significantly improved chemoresistance of U87 and A172 cells to TMZ (Fig. 4G, H). Next, we evaluated the tumorigenesis changes of GBM cells with PDLIM1-OE. By colony formation assays, we noticed a significant improvement in tumorigenesis with PDLIM1-OE (Fig. 4I–L). Meanwhile, we also performed soft agar assays and found that both the tumor sphere counts and diameters were significantly increased with PDLIM1-OE for both U87 and A172 cells (Fig. 4M–R). These results together suggested that PDLIM1-OE significantly improved the ratios of GSCs within CBM cell populations and thus promoted tumorigenesis and chemoresistance.

**Fig. 5:**
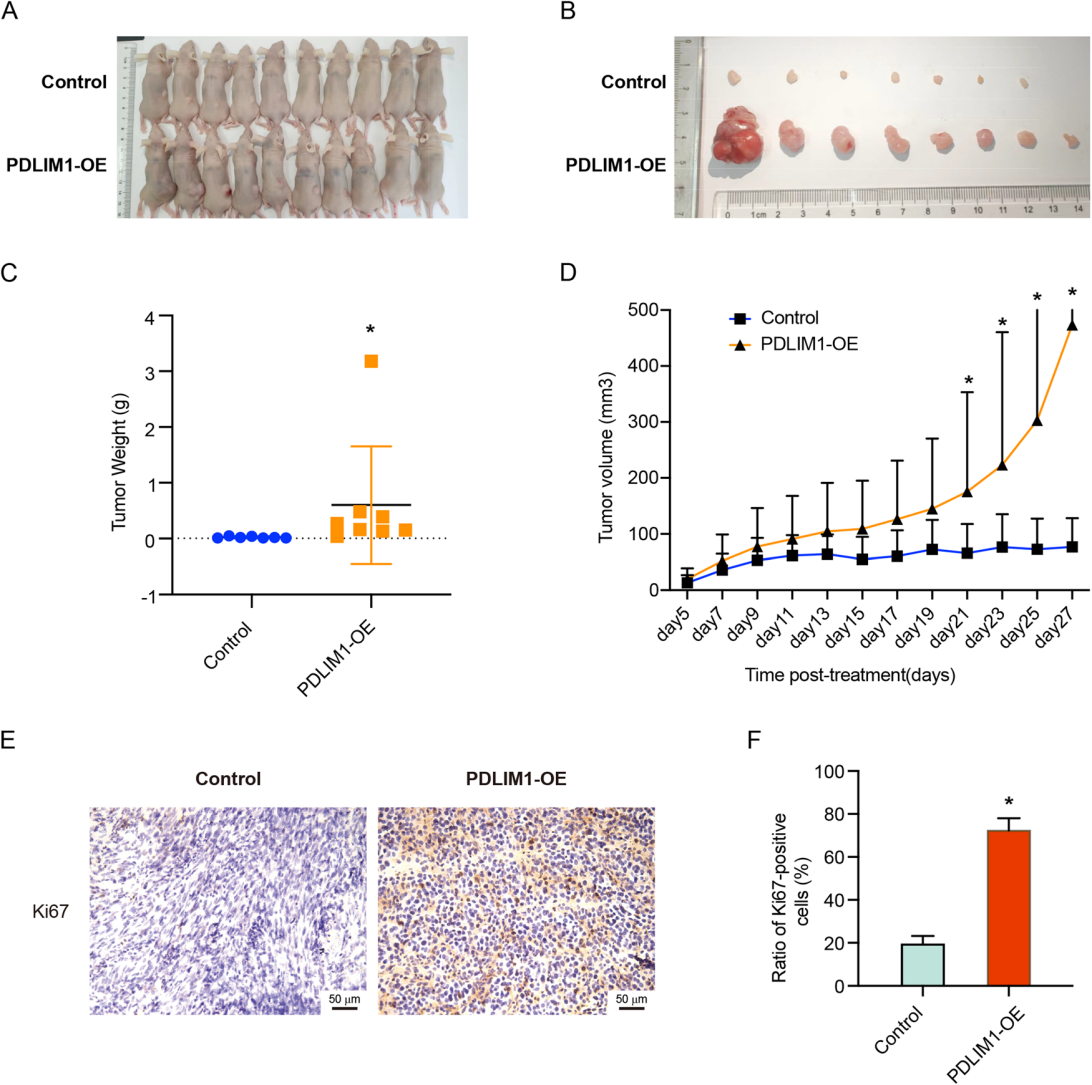
PDLIM1 overexpression promoted tumorigenesis in xenograft mice. Control and PDLIM1-overexpressing U87 cells were subcutaneously injected into BALB/c Nude Mice, respectively. Tumor growth was continuously monitored for 4 weeks. **A** The mice of both groups at sacrifice. **B** The tumors of both groups at sacrifice. **C** The weight of tumors in both groups. **D** The tumor volumes of both groups. **E**, **F** The immunohistochemistry staining against Ki67 on the tumor section slides of both groups. Control, empty vector control; PDLIM1-OE, PDLIM1 overexpression; *P < 0.05.

To further investigate the role of PDLIM1-OE in vivo, we performed xenograft tumor assays in SCID nude mice. By subcutaneous injection of WT and PDLIM1-OE U87 cells, we successfully generated tumors for both groups. Compared to the WT group, PDLIM1-OE led to more advanced tumor growth, as shown by the tumor weight and volume (Fig. 5A–D). By immunohistochemistry against Ki67 on the tumor slides of both the WT and PDLIM-OE groups, we noticed a significant upregulation of Ki67 in the PDLIM1-OE group, which was consistent with the results in vitro (Fig. 4E, F).

### PDLIM1 knockdown hindered GBM progression and GSC-mediated processes

**Fig. 6:**
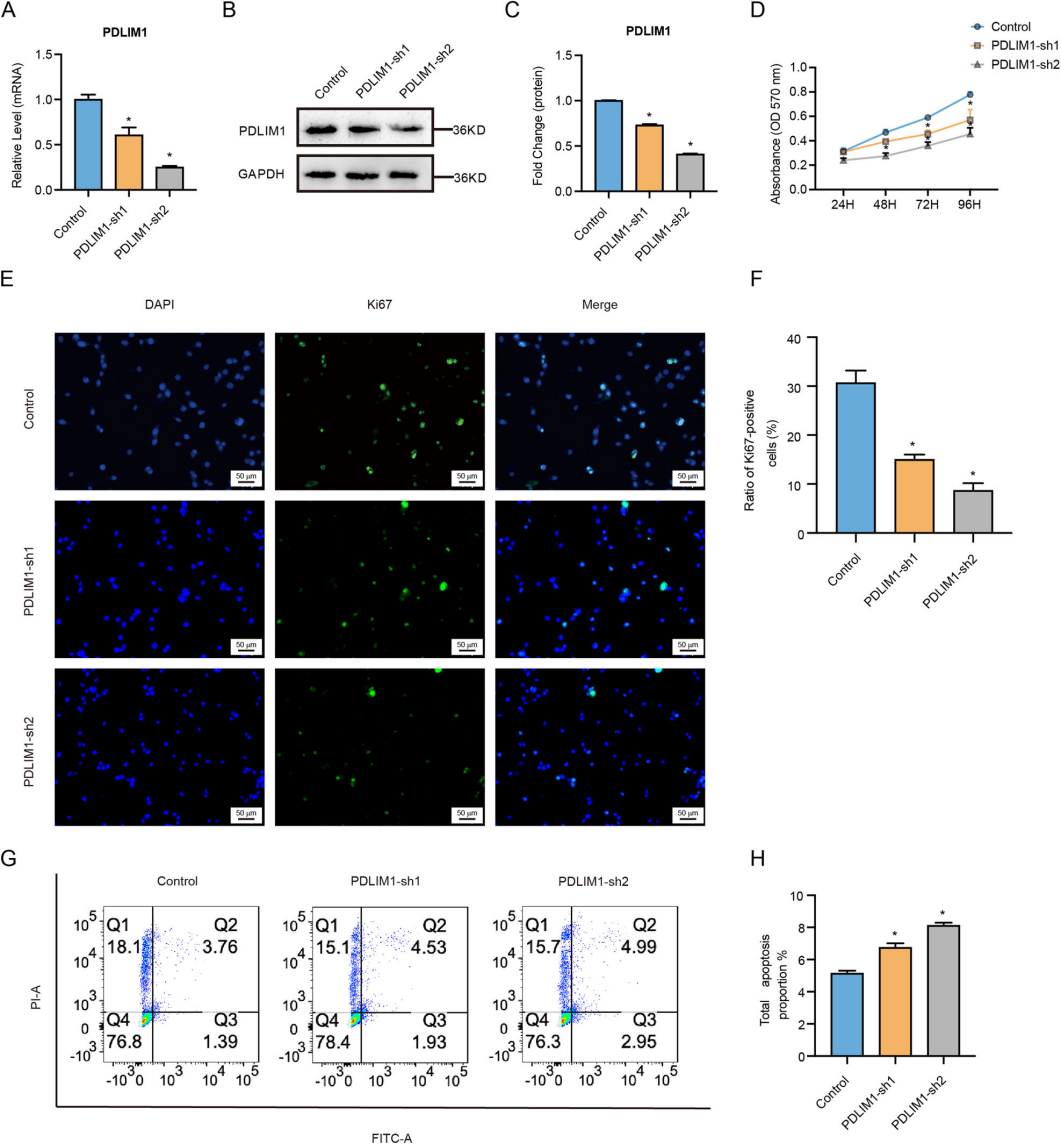
PDLIM1 knockdown inhibited GBM progression. The knockdown of PDLIM1 in U87 cells was validated by **A** RT-qPCR and **B**, **C** western blots. **D** MTT assays showing that PDLIM1 knockdown inhibited cell growth. **E**, **F** Immunostaining against Ki67 was performed on control and PDLIM1 knockdown U87 cells. **G**, **H** Annexin V/PI apoptosis assays were performed on control and PDLIM1 knockdown U87 cells. Control, scramble shRNA control; PDLIM1-sh1 and PDLIM1-sh2, two independent PDLIM1 knockdown; *P < 0.05.

We designed two independent shRNAs against PDLIM1 and transduced them into U87 cells to generate two PDLIM1 stably knockdown cell lines, PDLIM1-sh1 and PDLIM1-sh2. The scramble shRNA was also stably transduced into U87 cells as a control. The knockdown was verified using both RT-qPCR and western blots. Both PDLIM1-sh1 and PDLIM1-sh2 showed significant expression reductions in PDLIM1 compared to the scramble control, with the reduction extent more evident in PDLIM1-sh2 (Fig. 6A–C). By MTT assays, we noticed that both PDLIM1-sh1 and PDLIM1-sh2 experienced suppressed cell activities compared to the scramble control group, with the suppression extent of the PDLIM1-sh2 more evident (Fig. 6D). By immunostaining against Ki67, we noticed significant reductions of Ki67+ cell ratios in both PDLIM1 knockdown groups, with the PDLIM1-sh2 being of an even lower Ki67+ ratio (Fig. 6E, F). In contrast, the apoptosis was significantly promoted by PDLIM1 knockdown, with the PDLIM1-sh2 also displaying a higher apoptosis extent (Fig. 6G, H). These results together suggested that PDLIM1 was essential for GBM progression and its impact was dose-dependent.

**Fig. 7:**
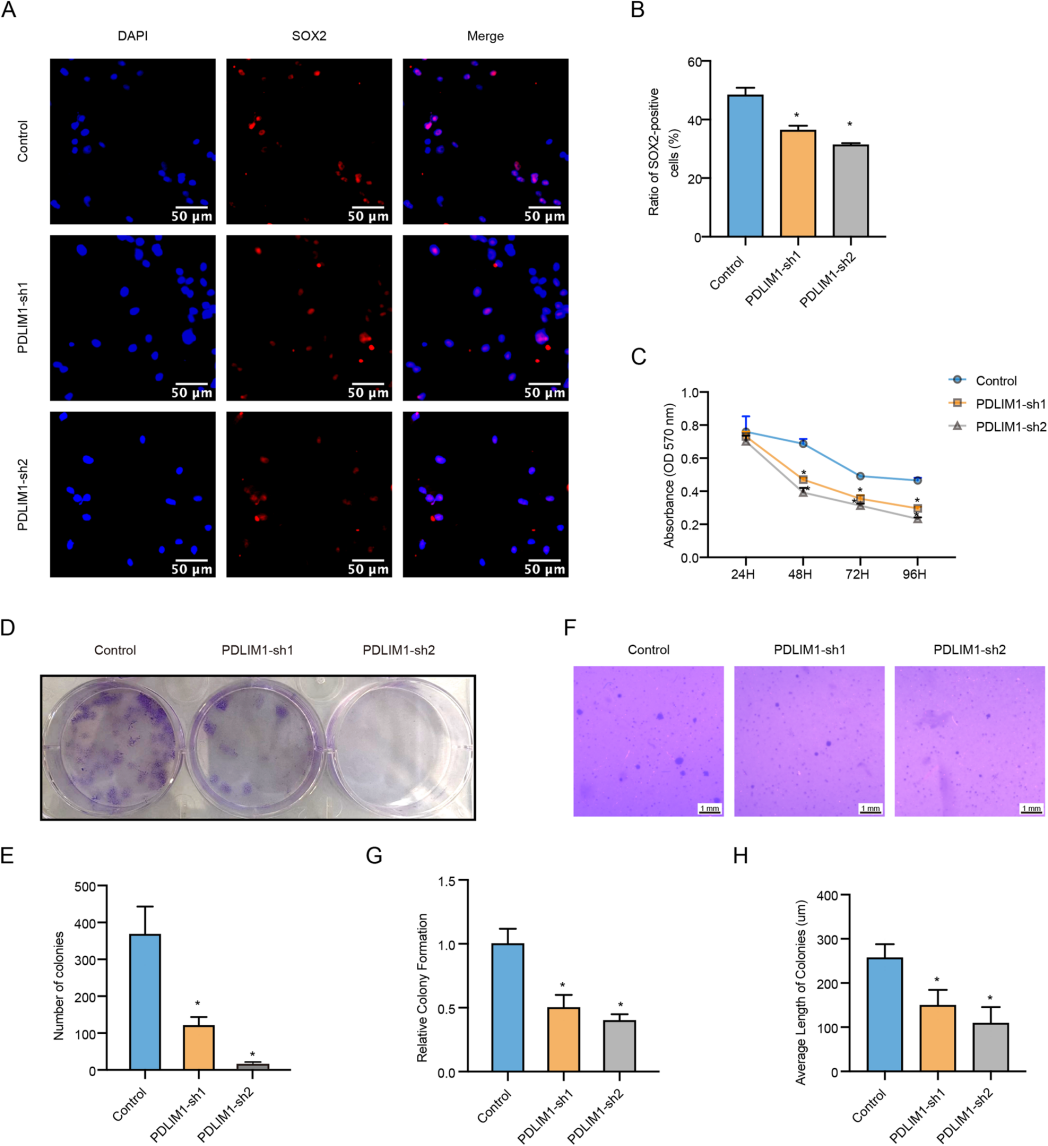
PDLIM1 knockdown inhibited GSC-mediated processes. **A**, **B** Immunostaining against SOX2 was performed on control and PDLIM1 knockdown U87 cells. **C** The knockdown of PDLIM1 significantly inhibited the chemoresistance of U87 cells to TMZ. **D**, **E** Colony formation assays were performed on control and PDLIM1 knockdown U87 cells. **F**–**H** Soft agar assays were performed on control and PDLIM1 knockdown U87 cells. Control, scramble shRNA control; PDLIM1-sh1 and PDLIM1-sh2, two independent PDLIM1 knockdown; *P < 0.05.

We next performed immunostaining against SOX2 on PDLIM1-sh1, PDLIM1-sh2, and scramble control U87 cells. As a result, both PDLIM1 knockdown U87 cells showed significant reductions in SOX2+ ratio, with the reduction extent being more evident in the PDLIM1-sh2, indicating that the maintenance of the GSCs within the GBM cell population required PDLIM1 (Fig. 7A, B). We then evaluated the chemoresistance changes regarding PDLIM1 knockdown. By treating PDLIM1-sh1, PDLIM-sh2, and scramble control U87 cells with TMZ, we found that PDLIM1 knockdown rendered GBM cells more sensitive to TMZ treatment (Fig. 7C). Through colony formation assays, we found that PDLIM1 knockdown significantly lowered the colony formation capabilities (Fig. 7D, E). Through soft agar assays, we found that PDLIM1 knockdown significantly reduced both colony count and size (Fig. 7F, H). Moreover, all these effects were in a dose-dependent manner. These results suggested that PDLIM1 knockdown significantly suppressed the maintenance of GSCs and thus hindered tumorigenesis and chemoresistance.

### PDLIM1 might regulate GBM progression and GSC through the PI3K-AKT pathway

**Fig. 8:**
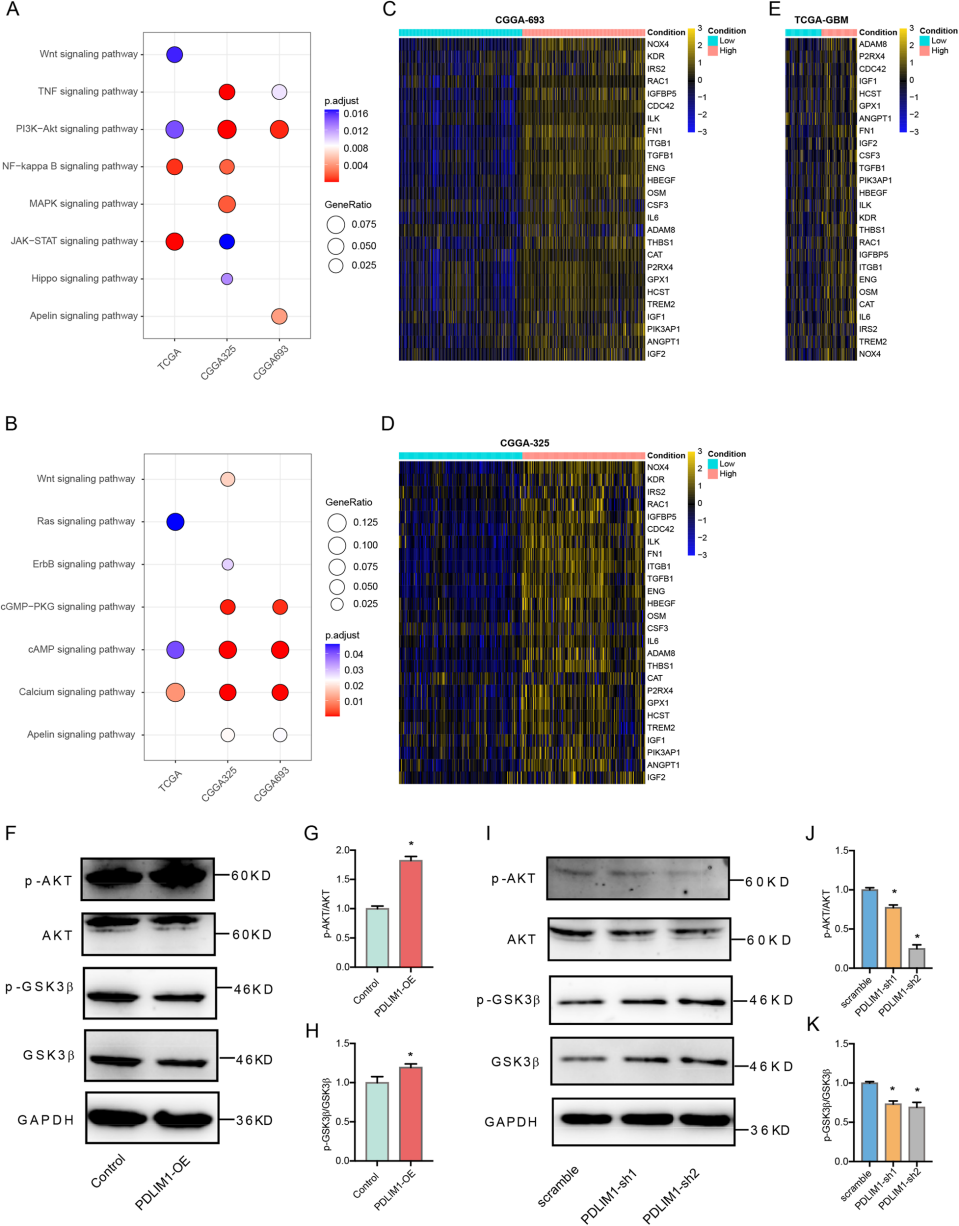
PDLIM1 might regulate GBM progression and GSC through the PI3K-AKT pathway. KEGG pathway analyses were performed on the **A** upregulated and **B** downregulated DEGs of the TCGA-GBM, CGGA-693, and CGGA-325 cohorts. The relative levels of PI3K-AKT pathway-related genes in low- and high-PDLIM1 expressing GBM samples of the **C** CGGA-693, **D** CGGA-325, and **E** TCGA-GBM cohorts. **F**–**H** Western blots against phosphorylated AKT (p-AKT), total AKT, phosphorylated GSK3β (p-GSK3β), and total GSK3β were performed on control and PDLIM1-overexpressing U87 cells. **I**–**K** Western blots against phosphorylated AKT (p-AKT), total AKT, phosphorylated GSK3β (p-GSK3β), and total GSK3β were performed on control and PDLIM1 knockdown U87 cells. GAPDH served as an internal control. Control, empty vector control; PDLIM1-OE, PDLIM1 overexpression; scramble, scramble shRNA control; PDLIM1-sh1 and PDLIM1-sh2, two independent PDLIM1 knockdown *P < 0.05.

To determine the downstream pathway that PDLIM1 might function through, we analyzed the bulk RNA-seq datasets of the TCGA-GBM, CGGA-693, and CGGA-325 cohorts, respectively. By setting the threshold of |log2(Fold Change)| > 1 and *P* < 0.05, we identified 533 upregulated and 84 downregulated DEGs for the TCGA-GBM cohort; 758 upregulated and 981 downregulated DEGs for the CGGA-693 cohort; 926 upregulated and 901 downregulated DEGS for the CGGA-325 cohort. By performing Kyoto Encyclopedia of Genes and Genomes (KEGG) pathway analyses on the above upregulated and downregulated DEGs, respectively, we found that the PI3K-AKT pathway was the only pathway significantly enriched in the upregulated DEGs of all three cohorts and the cAMP and Calcium signaling pathways were enriched in the downregulated DEGs of all three cohorts (Fig. 8A, B). The PI3K-AKT pathway was an established pathway that tightly controlled the tumorigenesis, GSC maintenance, and GSC-related functions in GBM [17, 18]. As such, we studied the expressions of the PI3K-AKT pathway-related genes in the RNA-seq data of all three cohorts. As a result, we found that the essential genes within the PI3K-AKT pathway were mutually upregulated in the high-PDLIM1 expression group (Fig. 8C–E).

We examined the changes in PI3K-AKT pathway activities with PDLIM1 overexpression and knockdown, respectively. By western blots, we found that the ratios of p-AKT/total AKT and p-GSK3β/GSK3β were significantly upregulated with PDLIM1 overexpression but suppressed with PDLIM1 knockdown in U87 cells (Fig. 8F–K). These results together implied that PDLIM1 might regulate GBM and GSC activities through manipulating the PI3K/AKT pathway.

## Discussion

Glioblastoma (GBM) is the most common and aggressive type of brain tumor with a poor prognosis despite numerous efforts to develop new therapeutic approaches. Tumors are highly heterogeneous in structure, consisting of different cell types, including cancer cells, immune cells, vascular cells, and cancer stem cells (GSCs). Among these cells, cancer stem cells (termed GSCs in GBM), are primarily responsible for the progression, recurrence, chemoresistance, and even metastasis [19, 20], making them a key target in GBM therapy. Therefore, there is an urgent need to discover novel GSC markers that can be used for diagnosis and drug targeting [21]. In this study, we integrated single-cell RNA sequencing (scRNA-seq) and bulk RNA-seq data to reveal that PDLIM1 is specifically expressed in GSCs and closely associated with GBM prognosis. In vitro and in vivo experiments further suggested that PDLIM1 regulates GBM progression and GSC-mediated activities, likely via the PI3K-AKT pathway.

scRNA-seq has been a powerful tool in deciphering the cellular composition of tissues, making it ideal for investigating the heterogeneity of tumors [22]. Before the advent of scRNA-seq, researchers either studied tumors as mixed cell populations or selectively sorted and purified a few cell types based on known markers, which inevitably missed certain valuable cell groups and failed to reveal their intrinsic gene profiles. Since its introduction, scRNA-seq has been applied to nearly all types of cancer. In this study, we analyzed a scRNA-seq dataset of GBM and annotated the GSCs among all GBM cells. We identified 1439 potential GSC marker genes by comparing GSCs to other GBM cells. Sequential analyses using univariate Cox regression and Log-Rank survival were performed on these potential markers in the TCGA-GBM cohort to select those significantly affecting GBM prognosis. Subsequently, we analyzed the expression of these selected markers across all GBM cell subgroups, aiming to further validate their specificity. Through these steps, we focused on PDLIM1, which was highly specifically expressed in GSCs and closely associated with GBM prognosis. This pipeline for discovering novel GSC markers was more efficient than traditional methods, benefiting from the advances in scRNA-seq.

GSCs have been a significant challenge in GBM therapy, and eliminating GSCs is believed to be key to improving the prognosis for GBM patients. Identifying specific GSC markers is a prerequisite for targeted therapy against GSCs. Many markers have been reported previously, such as PROM1, CD44, and SOX2 [5]. However, these markers are not always detectable or specific to GSCs under all circumstances [23]. Therefore, discovering more GSC markers, particularly those associated with GBM progression and prognosis, is necessary. Our data indicated that PDLIM1 is specifically expressed in GSCs among all GBM cell types. Moreover, the expression of other GSC markers correlated closely with PDLIM1. In scRNA-seq data, PDLIM1 correlated with markers such as PDGFRA, RBP1, and ITM2A. In bulk RNA-seq data, markers including CD44, PROM1, and SOX2 were significantly upregulated with high PDLIM1 expression. Co-immunostaining against PDLIM1 and SOX2 in PDLIM1-overexpressing and control U87 and A172 cells showed that PDLIM1 overexpression significantly increased the SOX2+ ratios, implying that PDLIM1 might promote the transition from non-GSCs to GSCs. Since GSCs are responsible for GBM metastasis and recurrence [19, 20], efficient suppression of GSC proportions in GBM might improve therapeutic outcomes. We found that PDLIM1 knockdown significantly reduced the GSC proportion in a dose-dependent manner. Colony formation and soft agar assays revealed that PDLIM1 overexpression enhanced tumorigenesis, while PDLIM1 knockdown had the opposite effect. Chemoresistance assays showed that PDLIM1 overexpression promoted GBM resistance to TMZ, while PDLIM1 knockdown repressed the chemoresistance. Since tumorigenesis and chemoresistance depend on GSCs, we believe that PDLIM1 may regulate these processes by adjusting GSC proportions among GBM cells. Meanwhile, we observed an increased proliferation observed in PDLIM1-overexpressing cells, as determined by the MTT assay and Ki67 immunostaining. Given that GSCs are often associated with a more quiescent or dormant phenotype in vivo, our findings suggested that PDLIM1 overexpression might drive a subset of GBM cells to exhibit both stem-like characteristics and higher proliferation rates. This highlights the adaptability of these cells under in vitro conditions, where they may not strictly adhere to the dormancy model typically observed in vivo.

PDLIM1 is a protein that usually functions as a scaffold for various factors, forming complexes. PDLIM1 has been reported to be involved in the regulation of multiple cancers, with distinct underlying mechanisms. In hepatocellular carcinoma, PDLIM1 competitively binds to ACTN4, disrupting the interaction between ACTN4 and F-actin, thereby activating the Hippo pathway and inhibiting metastasis [11]. In sarcoma, PDLIM1 regulates the Hippo pathway by inhibiting E3 ligase AIP-4, which mediates YAP1 degradation [24]. The interaction between PDLIM1 and the Wnt/β-catenin pathway is multifaceted. Several studies suggest that PDLIM1 plays a suppressive role in cancer progression. For example, in colorectal cancer, PDLIM1 facilitates the binding of β-catenin to E-cadherin, sequestering β-catenin in the cytoplasm, thereby inhibiting the Wnt/β-catenin pathway and suppressing tumor progression [25]. Similarly, PDLIM1 directly regulates Wnt3a expression, influencing cell migration and invasion in diabetic retinopathy [26]. However, other studies predominantly highlight PDLIM1’s promotive functions in cancer. PDLIM1 is regulated by miR-370-3p and influences the proliferation and apoptosis of chronic myelogenous leukemia (CML) cells through the Wnt/β-catenin pathway [8]. Additionally, PDLIM1 modulates the Warburg effect—a phenomenon where cancer cells exhibit increased glucose uptake and lactate production, critical for the tumor microenvironment—by interacting with HK2 and regulating the Wnt/β-catenin pathway in gastric cancer [27–29]. In breast cancer, PDLIM1 binds to α-actinin-4, activating CDC42 and promoting cell invasion and metastasis [12, 30]. In glioma, PDLIM1 was previously demonstrated to act as an adapter to p75NTR, driving glioma invasion [13]. However, the correlation between PDLIM1 and the PI3K-AKT pathway has been scarcely studied. Our analysis of RNA-seq data from three independent GBM cohorts indicated that the PI3K-AKT pathway was the only pathway mutually promoted with PDLIM1 upregulation. Western blots showed that the phosphorylation of AKT and GSK3β were promoted by PDLIM1 overexpression and suppressed by PDLIM1 knockdown in a dose-dependent manner. This finding is consistent with previous studies in breast cancer, where PDLIM1 knockdown inhibited p-AKT without affecting total AKT [12]. Extensive evidence has shown that the PI3K-AKT pathway is at least partially responsible for GSC maintenance and GSC-mediated processes [17, 18]. Therefore, we thought PDLIM1 might regulate GSC through the PI3K-AKT pathway, though the underlying molecular and pathway mechanisms warrant further investigation.

This study has several limitations. First, while our findings strongly suggest that PDLIM1 promotes GSC-like characteristics, further studies—such as assays for self-renewal and multipotency—are needed to fully explore its role in regulating the complete spectrum of stemness traits. Second, the effect of PDLIM1 on canonical stem cell markers, such as SOX2, was only investigated in vitro, and its impact in vivo remains to be determined. Third, our analysis of PDLIM1’s influence on the PI3K-AKT pathway was limited to examining the expression and phosphorylation levels of key pathway components. Future studies employing chemical activators or inhibitors of the PI3K-AKT pathway, as well as investigations into whether PDLIM1 regulates this pathway through its role in organizing the actin cytoskeleton, would further clarify the relationship between PDLIM1 and the PI3K-AKT pathway.

In conclusion, we identified a novel GSC marker, PDLIM1, which is highly specifically expressed in GSCs rather than other cell types within GBM. PDLIM1 was closely associated with GBM prognosis, tumorigenesis, and chemoresistance, potentially through manipulation of the PI3K-AKT pathway. Our study proposed PDLIM1 as a cell marker for GSCs and a potential therapeutic target in GBM, shedding light on targeted therapies against GSCs.

## Materials and methods

### Cell culture

U-87 MG (short for U87) and A172 cell lines were kindly provided by Cell Bank, Chinese Academy of Sciences. Both cells were authenticated by Short Tandem repeat profiling and free from mycoplasma contamination. Both cells were cultured with DMEM (Gibco) supplemented with 10% fetal bovine serum (FBS), 100 U/ml penicillin, and 100 µg/ml streptomycin (Biosharp). All cells were cultured in a humidified cell incubator with 5% CO_2_ at 37 °C.

For temozolomide (TMZ) treatment experiments, U87 and A172 cells were seeded onto 96-well plates at 3000 cells per well until they reached 80-90% confluence. To determine IC50 values, U87 cells were treated with TMZ at the concentrations of 0, 25, 50, 100, 200, 400, and 800 µM, and A172 cells were treated with TMZ at the concentrations of 0, 20, 40, 80, 160, and 320 µM. After 96 h, MTT assays were conducted (see “MTT Assay” section). IC50 values were calculated using GraphPad Prism 9. Cells were then treated with TMZ at their corresponding IC50 concentrations for 96 h, and MTT assays were performed every 24 h to monitor cell viability.

### Plasmids and transfection

PDLIM1-OE plasmid was constructed by ligating HA-tagged PDLIM1 ORF into the pcDNA3 vector at the EcoRI enzyme site. PDLIM1 knockdown and scramble control plasmids were constructed by ligating the designed PDLIM1 and scramble shRNAs into the pLL4.0 vector at the HpaI and XhoI enzyme sites, respectively. The sequences of synthesized primers and oligos are listed in Table S1.

Plasmid transfection was conducted using a transfection reagent, PolyJet (signaGen), following the manufacturer’s instructions. Cells used for plasmid transfections were plated 24 h prior to the transfections. The ratio of plasmids to the PolyJet used was 1:2 (μg:μL). The cell culture medium was changed 24 h post transfection. Stable cell lines were selected with 1.5 mg/mL G418 for pcDNA3-based plasmids and 1.5 µg/mL puromycin for pLL4.0-based plasmids.

### Animal studies

BALB/c Nude Mice (Jiangsu Qinglongshan Biotechnology Co. Ltd), aged 4–5 weeks, were randomly divided into control and PDLIM1-OE groups (*n* = 10 per group). Each group received a subcutaneous injection of 3.0 × 10^6^ control or PDLIM1-OE U87 cells. The width (W) and length (L) were measured with calipers bi-daily and tumor volume (*V*) was calculated using the formula: *V* = (*L* × *W*^2^)/2. After 4 weeks, mice were intraperitoneally administered with sodium pentobarbital (30 mg/kg) and euthanized by cervical dislocation. Primary tumors were excised and weighed before tumor volume reached 2000 mm^3^. No statistical methods to estimate sample size and no blinding was done.

### Immunohistochemical staining

Immunohistochemical staining was performed with an Immunohistochemistry Kit (Sangon) according to the manufacturer’s instructions. Paraffin-embedded slides were first dewaxed and then subjected to antigen retrieval with citrate solution in boiling water. Subsequently, the slides were blocked using bovine serum albumin (BSA) and incubated with primary antibodies overnight. On the next day, the slides were incubated with the kit’s built-in HRP-conjugated secondary antibodies and then reacted with the kit’s built-in DAB solution. Finally, slides were further stained with hematoxylin. The staining outcome was observed with a Leica DMi8 microscope. The antibodies used were as follows: PDLIM1 mAb (1:100, Santa Cruz Biotechnology, Cat# sc-393084), Ki67 mAb (1:100, Invitrogen, Cat# MA5-14520). This study was approved by the Ethics Committee of Anhui Normal University, and patient samples were collected with informed consent.

### Single-cell RNA sequencing (scRNA-seq) data analysis

The scRNA-seq data of GBM was downloaded from the Gene Expression Omnibus (GEO) database (GSE138794). The scRNA-seq data included the cells from 9 GBM patients, which were merged using the “CCA” package in R. Filtering criteria included 300–8000 features per cell, <20% mitochondrial genes, <22% ribosomal genes, and genes expressed in ≥3 cells. After the filtration, the scRNA-seq data consisted of 14,924 genes and 12,259 cells. The majority of scRNA-seq data analysis was performed using the “Seurat” package in R. Initially, the top 2000 altered genes were determined and applied to the principal component analysis (PCA). The top 30 principal components from the PCA were applied to the uniform manifold approximation and projection (UMAP). Cell clustering was performed using the “FindCluster” function in the “Seurat” package by setting the resolution at 0.5. Collectively, we obtained 25 cell clusters. The cell clusters were annotated with previously reported cell type markers and categorized into 9 cell types. The cell markers used were as follows: AQP4 and ALDH1L1 for astrocytes; MBP, PLP1, and ERMN for oligodendrocytes; TUG1 and MAP2 for neurons; CSPG4 for pericytes; GFAP for glial cells; C3 and DOCK8 for microglia cells; CD68 for macrophages; CD44 and PARP1 for cancer cells; PROM1, NES, and SOX2 for cancer stem cells. The potential markers of cancer stem cells were determined using the “FindMarkers” function with the significance cutoff of |logFC| > 0.25 and *P* < 0.05.

### MTT assay

MTT assays were conducted using the MTT assay kit (Sangon) according to the manufacturer’s instructions. Cells were seeded onto 96-well plates at a density of 2000 per well. The MTT assays were performed at 24, 48, 72, and 96 h post seeding. The signals of MTT assays were absorbance at 570 nm measured by a TECAN microplate reader.

### Cell apoptosis assay

Cell apoptosis was evaluated with an Annexin V/PI apoptosis kit (Sangon) according to the manufacturer’s instructions. Cells were trypsinized into single cells and resuspended with the kit’s built-in Binding Buffer. Subsequently, the cells were sequentially incubated with the kit built-in Annexin V-FITC for 15 min and PI for 30 min. Signals were measured with a flow cytometer (BD Biosciences).

### Immunostaining

Cell immunostaining was performed as reported previously [31]. Cells were fixed with 4% paraformaldehyde (PFA) and blocked with PBS containing 10% normal goat serum and 0.1% Triton X-100. The cells were then incubated with primary antibodies overnight. On the next day, the cells were briefly washed with PBS containing 0.1% Tween-20 and incubated with secondary antibodies for 90 minutes. Finally, the cells were stained with DAPI for 5 min. Staining outcomes were observed using a Leica DMi8 microscope. The antibodies used were as follows: SOX2 mAb (1:100, Santa Cruz Biotechnology, Cat# sc-365823), Ki67 mAb (1:100, Invitrogen, Cat# MA5-14520), HA Tag mAb (1:100, Invitrogen, Cat# 26183), Goat anti-Mouse IgG (H + L) Highly Cross-Adsorbed Secondary Antibody, Alexa Fluor Plus 555 (1:500, Invitrogen, Cat# A32727), Goat anti-Rabbit IgG (H + L) Cross-Adsorbed Secondary Antibody, Alexa Fluor 488 (1:500, Invitrogen, Cat# A11008).

### Western blots

Total protein was extracted using cell lysis buffer (Beyotime) supplemented with protease inhibitors (Roche) and phosphatase inhibitors (Beyotime). Protein concentrations were measured with a BCA protein assay kit (Biosharp) and adjusted to the same. Protein samples were subjected to electrophoresis on 10% SDS-PAGE gels and then transferred onto PVDF membranes. The membranes were then blocked with 5% BSA at room temperature for 1 hour and incubated with primary antibodies overnight at 4 °C. On the next day, the membranes were incubated with secondary antibodies for 1.5 h and then reacted with chemiluminescent substrates. The signals were observed using a Tanon 5200 imaging system and gel band intensities were determined using the ImageJ software. The antibodies used were as follows: PDLIM1 mAb (1:1000, Santa Cruz Biotechnology, Cat# sc-393084), AKT pAb (1:1000, Invitrogen, Cat# 44-609G), Phospho-AKT1 (Ser473) mAb (1:1000, Invitrogen, Cat# MA1-20325), GSK3β mAb (1:1000, Invitrogen, Cat# MA5-15109), Phospho-GSK-3β (Ser9) mAb (1:1000 Cell Signaling, Cat# 5558), GAPDH pAb (1:1000, Biosharp, Cat# BL006B), HRP-Conjugated Goat Anti-Mouse IgG (1:2000, Biosharp, Cat# BL001A), HRP-Conjugated Goat Anti-Rabbit IgG (1:2000, Biosharp, BL003A).

### Real-time quantitative PCR (RT-qPCR)

Total RNA was extracted with a total RNA isolation reagent (Biosharp). Reverse transcriptions were performed on the total RNA with the MonScript RTIII All-in-One Mix with dsDNase Kit (Monad). Subsequently, the quantitative PCRs were performed with MonAmp TM ChemoHS qPCR Mix (Monad). These experiments were conducted according to their corresponding manufacturer’s instructions. GAPDH served as an internal control. The sequences of RT-qPCR primers are listed in Table S2.

### Colony formation assay

For colony formation assays, 15,000 cells were seeded onto 6-well plates and cultured for 14 days. The medium was changed every two days. At the end of the assays, the cells were washed with PBS and stained with 0.01% crystal violet for 20 min. The staining outcomes were photographed and the colony numbers were counted.

### Soft agar assay

Soft agar assays were performed in 6-well plates that were pre-coated with 0.6% agarose. Cells were suspended with 0.5% agarose in complete culture medium and then plated onto the 0.6% agarose-coated plates. The soft agar culture system was soaked in the complete culture medium and cultured until the colonies were visible. The whole system was then stained with 0.01% crystal violet. The colony numbers and diameters were recorded and analyzed.

### Bulk RNA sequencing (RNA-seq) data analysis

In this study, we downloaded and analyzed three independent cohorts of RNA-seq data, which were the TCGA-GBM, CGGA-693, and CGGA-325. The TCGA-GBM cohort was downloaded from The Cancer Genome Atlas (TCGA) database, including 169 GBM samples and 5 normal tissues. The CGGA-693 and CGGA-325 cohorts were both downloaded from the Chinese Glioma Genome Atlas (CGGA) database, including 693 and 325 GBM samples, respectively. Each cohort was divided into low- and high-PDLIM1 groups according to their corresponding median expressions of PDLIM1. The clinical information is shown in Tables S3~S5. Differentially expressed genes (DEGs) between the low- and high-PDLIM1 groups were determined using the DESeq2 package in R. The significance cutoff was *P* < 0.05 and |log2(FoldChange)| > 1. Kyoto Encyclopedia of Genes and Genomes (KEGG) pathway analysis was conducted using the ClusterProfiler package in R using the significance cutoff of *P* < 0.05.

### Kaplan–Meier survival analysis

The patients in the TCGA-GBM, CGGA-693, and CGGA-325 cohorts were divided into low- and high-PDLIM1 groups. The overall survival (OS) status and intervals of all patients were incorporated with their corresponding PDLIM1 expressions. Kaplan–Meier survival analysis was performed using the survival and survminer packages in R. *P* < 0.05 was considered significant.

### Cox regression analysis

The clinical information of the TCGA-GBM, CGGA-693, and CGGA-325 cohorts was incorporated with the PDLIM1 expressions. The clinical information of the CGGA-693 and CGGA-325 cohorts included recurrent status, gender, age, IDH1 mutation, and histological stage, while the TCGA-GBM cohort only provided the gender, race, and age information. Both univariant and multivariant Cox regression analyses were performed using the survival package in R. *P* < 0.05 was considered significant.

### Data analysis

All experiments were biologically repeated at least three times. Student’s *t*-test was conducted for two-group comparisons, and one-way analysis of variance (ANOVA) was conducted for three or more group comparisons. *P* < 0.05 was considered to be statistically significant. All data were presented as mean ± SD.

## Supplementary information

Supplementary figure legends

Figure S1

Figure S2

Table S1

Table S2

Table S3

Table S4

Table S5

Original western blot images

## Data Availability

Bulk RNA-seq data of GBM and clinical information were downloaded from The Cancer Genome Atlas (TCGA) (the TCGA-GBM cohort) and the Chinese Glioma Genome Atlas (CGGA) database (the CGGA-693 and CGGA-325 cohorts). scRNA-seq data of GBM was downloaded from the GEO database (GSE138794).
