# Supplementary material for: Identification of PDLIM1 as a glioblastoma stem cell marker driving tumorigenesis and chemoresistance
Source: Cell Death Discov. 2024 Nov 15;10:469. doi: 10.1038/s41420-024-02241-7 (PMC11568334; doi:10.1038/s41420-024-02241-7)
Supplement: Supplementary file 1 — Supplementary figure legends [file 41420_2024_2241_MOESM1_ESM.docx]

# Supplementary figure legends

**Figure S1. PDLIM1 was closely associated with GSCs in GBM.** (**A**) The chord diagram showing the correlation between PDLIM1 and all cell subgroups in GBM. (**B**) The chord diagram showing the correlation between the marker genes of all cell subgroups in GBM. The relative levels of GSC-related genes in low- and high-PDLIM1 expressing GBM samples of the (**C**) CGGA-693, (**D**) CGGA-325, and (**E**) TCGA-GBM cohorts.

**Figure S2. PDLIM1 was an independent prognostic factor for GBM.** (**A**) Univariant and (**B**) multivariant Cox regression analyses were performed on PDLIM1 expressions and other clinical parameters in the CGGA-693 cohort. (**C**) The distributions of PDLIM1 expressions, vital status, and OS in the high- and low-risk groups of the CGGA-693 cohort. The risk scores were calculated with the survival package in R. (**D**) Univariant and (**E**) multivariant Cox regression analyses were performed on PDLIM1 expressions and other clinical parameters in the CGGA-325 cohort. (**F**) The distributions of PDLIM1 expressions, vital status, and OS in the high- and low-risk groups of the CGGA-325 cohort. The risk scores were calculated with the survival package in R. (**G**) Univariant and (**H**) multivariant Cox regression analyses were performed on PDLIM1 expressions and other clinical parameters in the TCGA-GBM cohort. (**I**) The distributions of PDLIM1 expressions, vital status, and OS in the high- and low-risk groups of the TCGA-GBM cohort. The risk scores were calculated with the survival package in R.
