# Supplementary figures and images for: Identification of PDLIM1 as a glioblastoma stem cell marker driving tumorigenesis and chemoresistance
Source: Cell Death Discov. 2024 Nov 15;10:469. doi: 10.1038/s41420-024-02241-7 (PMC11568334; doi:10.1038/s41420-024-02241-7)

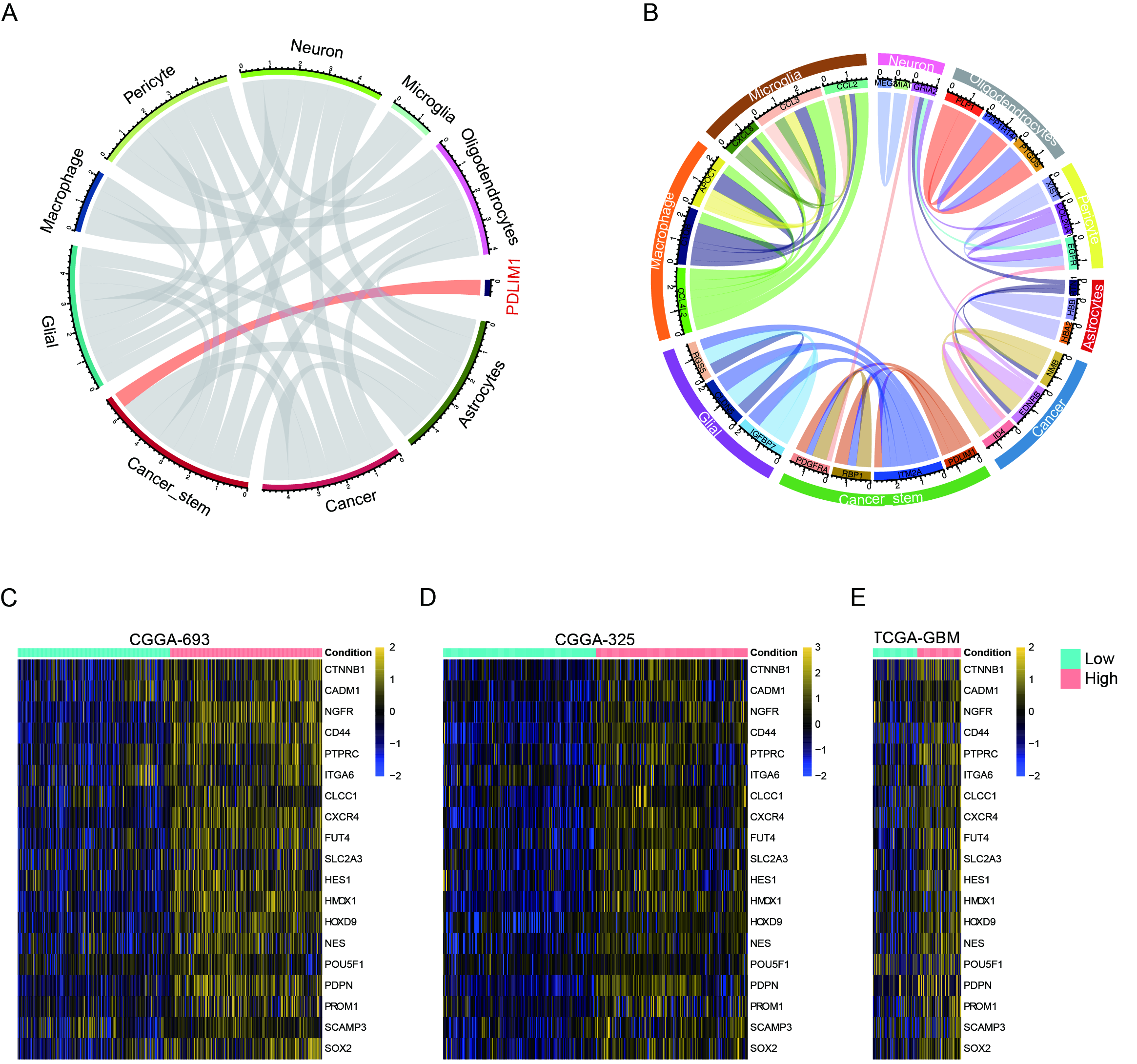

Supplement: Supplementary file 2 — Figure S1 [file 41420_2024_2241_MOESM2_ESM.tif]

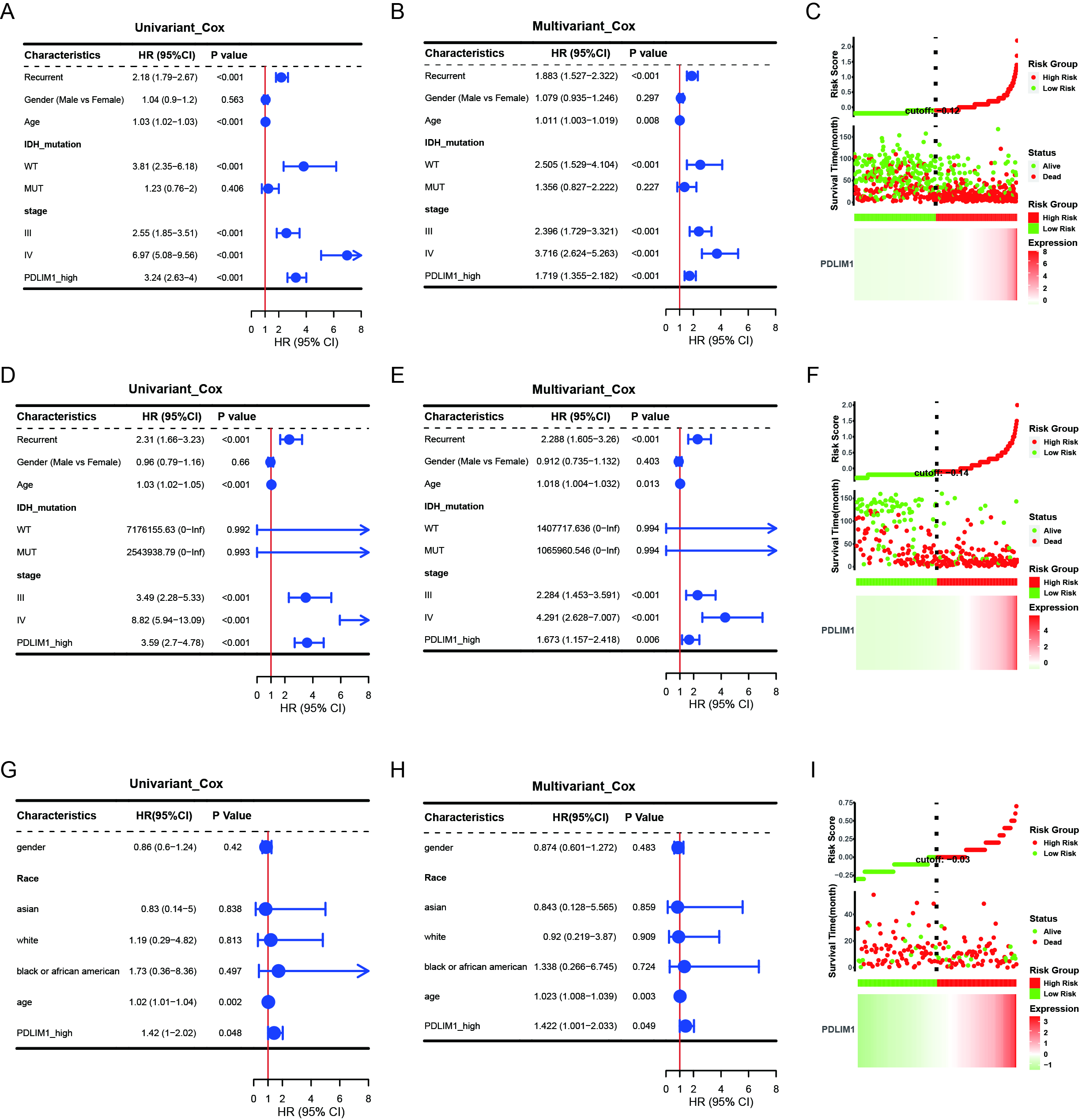

Supplement: Supplementary file 3 — Figure S2 [file 41420_2024_2241_MOESM3_ESM.tif]
