# Supplementary material for: Identification of PDLIM1 as a glioblastoma stem cell marker driving tumorigenesis and chemoresistance
Source: Cell Death Discov. 2024 Nov 15;10:469. doi: 10.1038/s41420-024-02241-7 (PMC11568334; doi:10.1038/s41420-024-02241-7)
Supplement: Supplementary file 4 — Table S1 [file 41420_2024_2241_MOESM4_ESM.docx]

**Table S1** The sequences of synthesized primers and oligos

| **Name** | **Sequence** |
| --- | --- |
| PDLIM1-OE-F | CCGAATTCGCCACCATGACCACCCAGCAGATAGAC |
| PDLIM1-OE-R | CGCGAATTCTCACTAAGCGTAATCTGGAACATCGTATGGGTACTTGGGGAACACAGTGACCA |
| shPDLIM1-1-s | TGCCTTGGTTAATTGACTCACATTCAAGAGATGTGAGTCAATTAACCAAGGCTTTTTTC |
| shPDLIM1-1-as | TCGAGAAAAAAGCCTTGGTTAATTGACTCACATCTCTTGAATGTGAGTCAATTAACCAAGGCA |
| shPDLIM1-2-s | TGCTCAGAAGTTGCCTATGTGTTTCAAGAGAACACATAGGCAACTTCTGAGCTTTTTTC |
| shPDLIM1-2-as | TCGAGAAAAAAGCTCAGAAGTTGCCTATGTGTTCTCTTGAAACACATAGGCAACTTCTGAGCA |
| shscramble-s | TGGGTGAACTCACGTCAGAATTCAAGAGATTCTGACGTGAGTTCACCCTTTTTTC |
| shscramble-as | TCGAGAAAAAAGGGTGAACTCACGTCAGAATCTCTTGAATTCTGACGTGAGTTCACCCA |
