# Supplementary material for: Identification of PDLIM1 as a glioblastoma stem cell marker driving tumorigenesis and chemoresistance
Source: Cell Death Discov. 2024 Nov 15;10:469. doi: 10.1038/s41420-024-02241-7 (PMC11568334; doi:10.1038/s41420-024-02241-7)
Supplement: Supplementary file 5 — Table S2 [file 41420_2024_2241_MOESM5_ESM.docx]

**Table S2** The sequences of RT-qPCR primers

| **Name** | **Sequence** |
| --- | --- |
| PDLIM1-F | GTGGAGGCGAACAGCAGACC |
| PDLIM1-R | CCAATCGACGCAGCCACTTT |
| hGAPDH-F | ACCATCTTCCAGGAGCGAGAT |
| hGAPDH-R | ATGACGAACATGGGGGCATC |
