# Supplementary material for: Identification of PDLIM1 as a glioblastoma stem cell marker driving tumorigenesis and chemoresistance
Source: Cell Death Discov. 2024 Nov 15;10:469. doi: 10.1038/s41420-024-02241-7 (PMC11568334; doi:10.1038/s41420-024-02241-7)
Supplement: Supplementary file 6 — Table S3 [file 41420_2024_2241_MOESM6_ESM.docx]

**Table S3** Clinical characteristics of the TCGA-GBM cohort

|  | **PDLIM1_high (N=81)** | **PDLIM1_low (N=81)** | **P-value** |
| --- | --- | --- | --- |
| **gender** |  |  |  |
| MALE | 56 (69.1%) | 48 (59.3%) | 0.251 |
| FEMALE | 25 (30.9%) | 33 (40.7%) |  |
| **race** |  |  |  |
| asian | 2 (2.5%) | 3 (3.7%) | 0.509 |
| white | 73 (90.1%) | 71 (87.7%) |  |
| black or african american | 6 (7.4%) | 5 (6.2%) |  |
| not reported | 0 (0%) | 2 (2.5%) |  |
| **AGE** |  |  |  |
| <=60 | 40 (49.4%) | 43 (53.1%) | 0.753 |
| >60 | 41 (50.6%) | 38 (46.9%) |  |
| **PDLIM1** |  |  |  |
| Mean (SD) | 56.1 (21.1) | 19.8 (8.03) | <0.001 |
| Median [Min, Max] | 51.5 [32.4, 152] | 20.6 [2.55, 32.3] |  |

Note: patients without prognostic information were excluded from the study.
