# Supplementary material for: Identification of PDLIM1 as a glioblastoma stem cell marker driving tumorigenesis and chemoresistance
Source: Cell Death Discov. 2024 Nov 15;10:469. doi: 10.1038/s41420-024-02241-7 (PMC11568334; doi:10.1038/s41420-024-02241-7)
Supplement: Supplementary file 7 — Table S4 [file 41420_2024_2241_MOESM7_ESM.docx]

**Table S4** Clinical characteristics of the CGGA-693 cohort

|  | **PDLIM1_low (N=329)** | **PDLIM1_high (N=328)** | **P-value** |
| --- | --- | --- | --- |
| **PRS_type** |  |  |  |
| Primary | 248 (75.4%) | 156 (47.6%) | <0.001 |
| Recurrent | 81 (24.6%) | 172 (52.4%) |  |
| **Gender** |  |  |  |
| MALE | 187 (56.8%) | 187 (57.0%) | 1 |
| FEMALE | 142 (43.2%) | 141 (43.0%) |  |
| **IDH_mutation** |  |  |  |
| WT | 72 (21.9%) | 204 (62.2%) | <0.001 |
| MUT | 221 (67.2%) | 112 (34.1%) |  |
| NA | 36 (10.9%) | 12 (3.7%) |  |
| **stage** |  |  |  |
| II | 125 (38.0%) | 47 (14.3%) | <0.001 |
| III | 150 (45.6%) | 98 (29.9%) |  |
| IV | 54 (16.4%) | 183 (55.8%) |  |
| **AGE** |  |  |  |
| <=45 | 216 (65.7%) | 175 (53.4%) | 0.00401 |
| >45 | 113 (34.3%) | 152 (46.3%) |  |
| NA | 0 (0%) | 1 (0.3%) |  |
| **vital_status** |  |  |  |
| ALIVE | 189 (57.4%) | 74 (22.6%) | <0.001 |
| DEAD | 140 (42.6%) | 254 (77.4%) |  |
| **PDLIM1** |  |  |  |
| Mean (SD) | 2.61 (1.57) | 29.0 (27.0) | <0.001 |
| Median [Min, Max] | 2.49 [0, 5.96] | 20.9 [6.01, 203] |  |

Note: patients without prognostic information were excluded from the study.
