# Supplementary material for: Identification of PDLIM1 as a glioblastoma stem cell marker driving tumorigenesis and chemoresistance
Source: Cell Death Discov. 2024 Nov 15;10:469. doi: 10.1038/s41420-024-02241-7 (PMC11568334; doi:10.1038/s41420-024-02241-7)
Supplement: Supplementary file 8 — Table S5 [file 41420_2024_2241_MOESM8_ESM.docx]

**Table S5** Clinical characteristics of the CGGA-325 cohort

|  | **PDLIM1_low (N=154)** | **PDLIM1_high (N=159)** | **P-value** |
| --- | --- | --- | --- |
| **PRS_type** |  |  |  |
| Primary | 118 (76.6%) | 104 (65.4%) | 0.0248 |
| Recurrent | 36 (23.4%) | 51 (32.1%) |  |
| NA | 0 (0%) | 4 (2.5%) |  |
| **Gender** |  |  |  |
| MALE | 89 (57.8%) | 108 (67.9%) | 0.0821 |
| FEMALE | 65 (42.2%) | 51 (32.1%) |  |
| **IDH_mutation** |  |  |  |
| WT | 27 (17.5%) | 118 (74.2%) | <0.001 |
| MUT | 126 (81.8%) | 41 (25.8%) |  |
| NA | 1 (0.6%) | 0 (0%) |  |
| **stage** |  |  |  |
| II | 86 (55.8%) | 12 (7.5%) | <0.001 |
| III | 42 (27.3%) | 32 (20.1%) |  |
| IV | 26 (16.9%) | 111 (69.8%) |  |
| Missing | 0 (0%) | 4 (2.5%) |  |
| **AGE** |  |  |  |
| <=45 | 118 (76.6%) | 75 (47.2%) | <0.001 |
| >45 | 36 (23.4%) | 84 (52.8%) |  |
| **vital_status** |  |  |  |
| ALIVE | 72 (46.8%) | 23 (14.5%) | <0.001 |
| DEAD | 82 (53.2%) | 136 (85.5%) |  |
| **PDLIM1** |  |  |  |
| Mean (SD) | 3.45 (2.16) | 31.8 (24.8) | <0.001 |
| Median [Min, Max] | 2.91 [0.620, 8.03] | 25.1 [8.06, 150] |  |

Note: patients without prognostic information were excluded from the study.
