# Supplementary material for: Identification of PDLIM1 as a glioblastoma stem cell marker driving tumorigenesis and chemoresistance
Source: Cell Death Discov. 2024 Nov 15;10:469. doi: 10.1038/s41420-024-02241-7 (PMC11568334; doi:10.1038/s41420-024-02241-7)
Supplement: Supplementary file 9 — Original western blot images [file 41420_2024_2241_MOESM9_ESM.pdf]

A

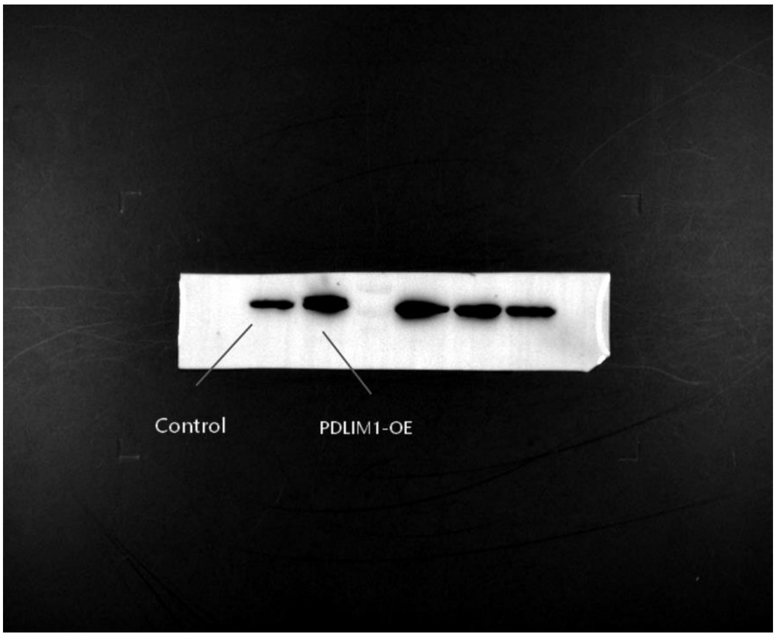

B

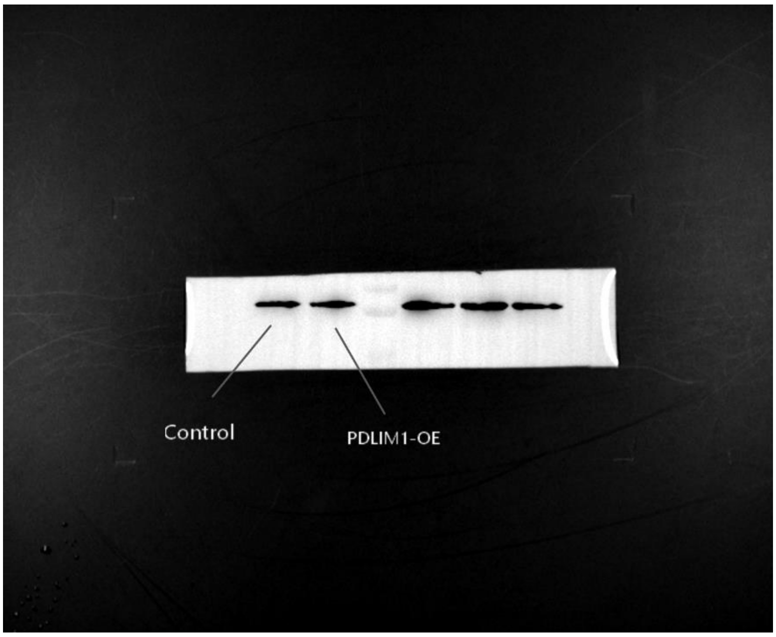

Original western blot images for Figure 3B. (**A**) The original images for the PDLIM1 blot. (**B**) The original images for the GAPDH blot. The “Control” and “PDLIM1-OE” lanes were labeled in these original images.

A

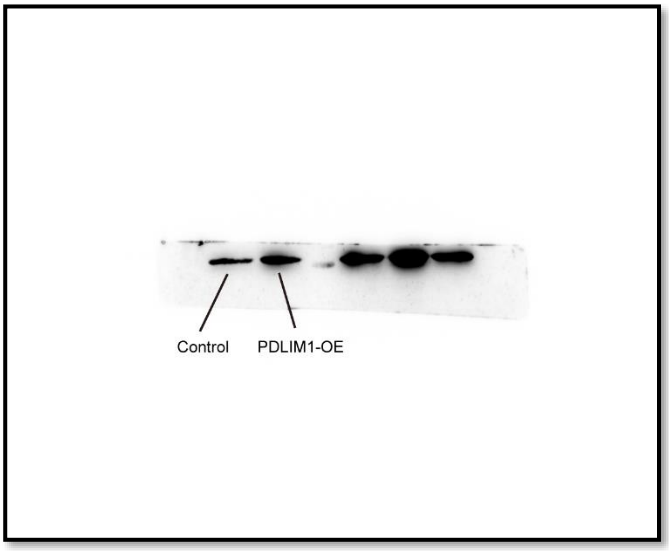

B

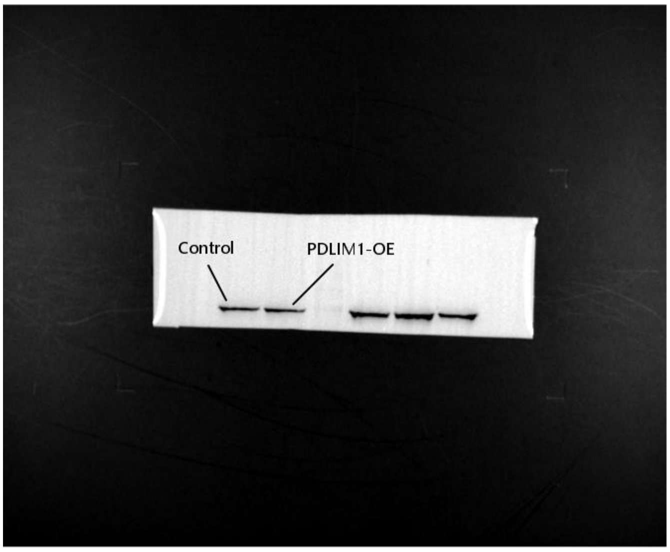

Original western blot images for Figure 3E. (**A**) The original images for the PDLIM1 blot. (**B**) The original images for the GAPDH blot. The "Control" and "PDLIM1-OE" lanes were labeled in these original images.

A

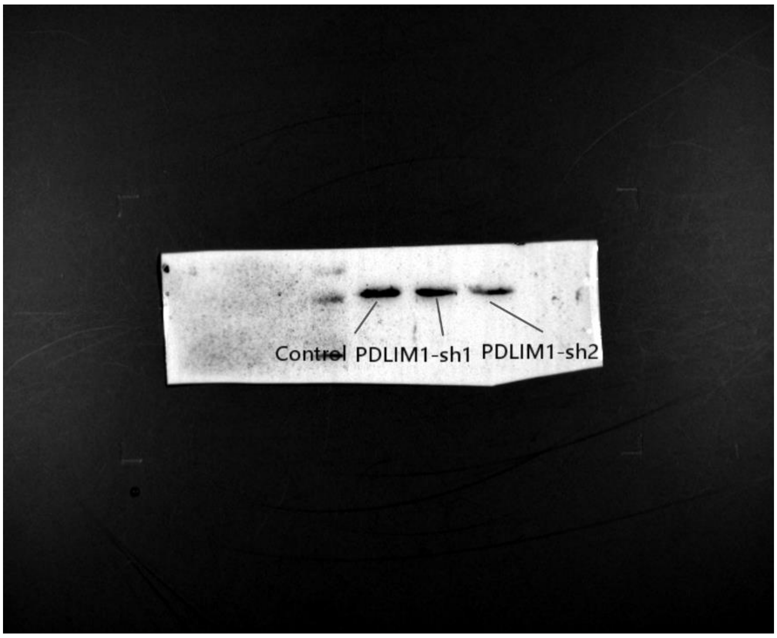

B

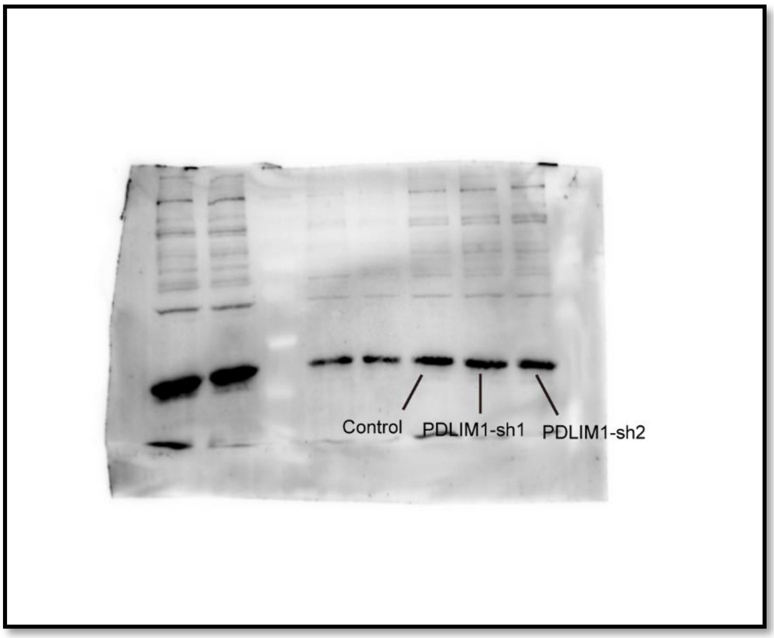

Original western blot images for Figure 6B. (**A**) The original images for the PDLIM1 blot. (**B**) The original images for the GAPDH blot. The “Control”, “PDLIM1-sh1”, and “PDLIM1-sh2” lanes were labeled in these original images.

A

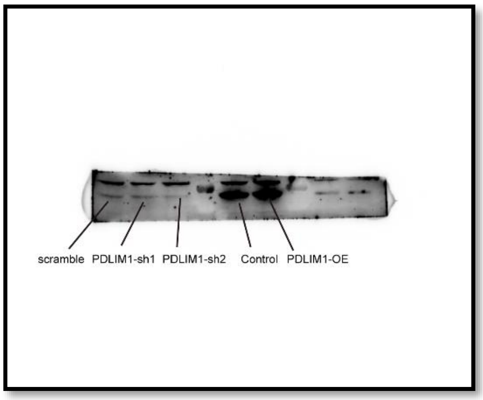
scramble
PDLIM1-sh1
PDLIM1-sh2
Control
PDLIM1-OE

B

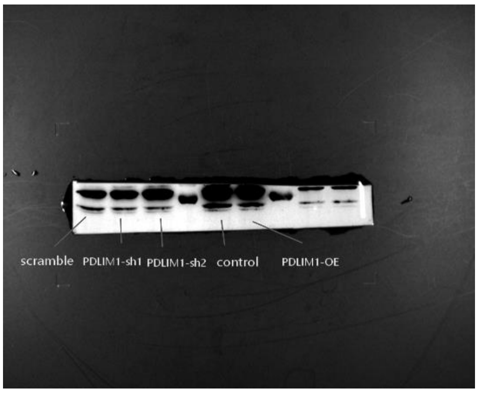
scramble
PDLIM1-sh1
PDLIM1-sh2
control
PDLIM1-OE

C

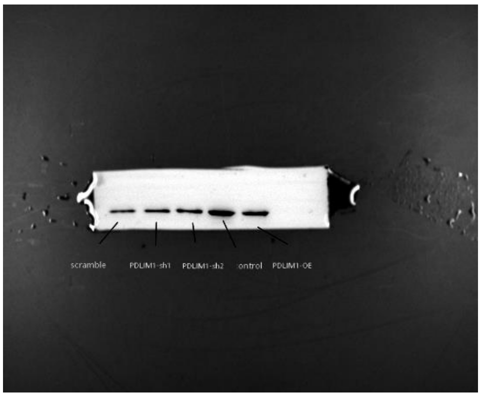
scramble
PDLIM1-sh1
PDLIM1-sh2
ontrol
PDLIM1-OE

D

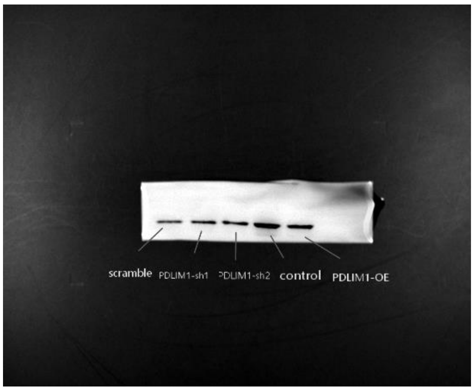
scramble
PDLIM1-sh1
PDLIM1-sh2
control
PDLIM1-OE

E

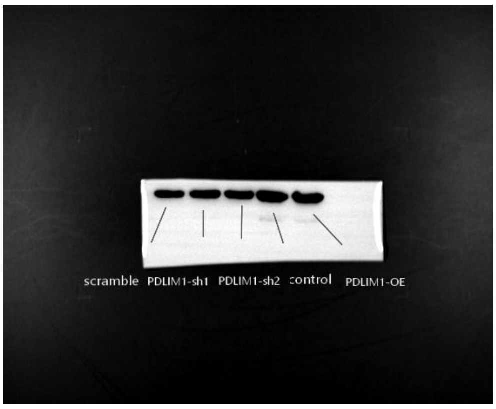

Original western blot images for Figure 8. The western blots in Figure 8F and Figure 8I were performed on one PAGE gel and PVDF membrane. Therefore, the original images here included both groups. (**A**) The original images for the p-AKT blots. (**B**) The original images for the total AKT blots. (**C**) The original images for the p-GSK3β blots. (**D**) The original images for the GSK3β blots. (**E**) The original images for the GAPDH blots. The “scramble”, “PDLIM1-sh1”, “PDLIM1-sh2”, “Control” and “PDLIM1-OE” lanes were labeled in these original images.
